# Treatment of Xenobiotic Cyclic Nitramine Explosives in Wastewater

**DOI:** 10.3390/jox15060188

**Published:** 2025-11-07

**Authors:** Swati Gupta, Zeev Ronen

**Affiliations:** Department of Environmental Hydrology and Microbiology, The Zuckerberg Institute for Water Research, The Jacob Blauste Institute for Desert Research, Ben-Gurion University of the Negev, Midreshet Ben-Gurion 8499000, Israel; guptas@post.bgu.ac.il

**Keywords:** nitramine, RDX, wastewater, HMX, aerobic degradation, contaminants, CL-20, microbial degradation, toxicity

## Abstract

Cyclic nitramine explosives such as octahydro-1,3,5,7-tetranitro-1,3,5,7-tetrazocine (HMX), hexahydro-1,3,5-trinitro-1,3,5-triazine (RDX), and 2,4,6,8,10,12-hexanitro-2,4,6,8,10,12-hexaazaisowurtzitane (CL-20) are xenobiotics that are utilized in a variety of propellants and traditional weapons. The primary source of water contamination is the industrial use of these hazardous substances in propellants and wastewater generated from munitions production facilities. These chemicals have a negative impact on human health and ecosystems. It is necessary to remove these toxic compounds from the environment safely because their production and usage have seriously contaminated soil and groundwater. Although there are no widely adopted WHO or US federal Maximum Contaminant Levels (MCLs) for military explosives, the health advisory limits for RDX in drinking water are 2 µg/L, and for HMX are 400 µg/L. Numerous traditional treatment approaches that incorporate physical, biological, and chemical processes have been used to decontaminate explosive wastewater. However, contaminants are not completely mineralized by these methods. Complete reduction of these chemicals can be accomplished by combining suitable methods. For the remediation of explosive effluent, integrated treatment systems that combine the effectiveness of biological and physical-chemical methods have shown promising results. This review discusses the toxicity and some physical–chemical–biological and combined treatment processes of wastewater polluted by these explosive contaminants.

## 1. Introduction

Water is essential to life and the balance of ecosystems; yet, pollution poses significant risks to it. Significant volumes of industrial effluents are released into the water system by industries such as pharmaceuticals, pulp and paper, textiles, leather, and explosives. Xenobiotic chemicals, such as explosive waste, now pose a threat to living things worldwide due to their environmental persistence. Explosives are highly reactive compounds that, when oxidized, produce harmful substances in the surrounding environment. Incomplete rocket and bomb detonations pollute military training facilities [1,2].

In contrast to incomplete detonations, which distribute explosives throughout impact areas, unexploded ordnance releases chemicals exclusively. Many of the explosives-contaminated sites have been identified in the United States, Europe, Canada, and Asia. The waste generated from production, dumping, and military operations is a source point for explosives into aquatic habitats [3,4,5,6,7]. According to investigations conducted on military ranges in the United States and Canada, leftover explosives can be extensively distributed throughout the soil. These leftovers can enter groundwater and surface water and affect the environment [8]. Depending on chemical properties, explosive waste can be hazardous and persistent, damaging ecosystems by getting into food chains [9]. The three primary types of significant explosives are nitramines, nitroaromatics, and nitrate esters. Globally, cyclic nitramine explosives are the most produced as military weapons, and their use has contaminated the environment. Three frequently utilized cyclic nitramine explosives are octahydro-1,3,5,7-tetranitro-1,3,5,7-tetrazocine, or High Melting Explosive (HMX), or Octogen, hexahydro-1,3,5-trinitro-1,3,5-triazine, or Research Department Explosive (RDX), or Cyclonite, and 2,4,6,8,10,12-hexanitro-2,4,6,8,10,12-hexaazaisowurtzitane (CL-20) [2,8,10].

These nitrogen-based compounds are extremely energetic and release a significant amount of energy when they break down in excess. Since nitramines are categorized as secondary explosives, the primary explosive is necessary to initiate them because they cannot be easily detonated [11]. HMX and RDX are extensively produced as defense weapons worldwide and utilized as chemical explosives due to their better stability and detonation intensity. Both compounds are synthetic and are not naturally occurring [12]. These chemicals have been widely utilized in various military activities during World War II and current military conflicts around the globe. Both explosives have been designated as priority pollutants and contaminants of concern, respectively, by the US Environmental Protection Agency (2004) [13]. Globally, the extensive usage of RDX and HMX has seriously contaminated the surrounding environment [14]. RDX and HMX are anticipated to be replaced by CL-20, a potent new energetic explosive with moderate sensitivity. The energy and density of CL-20 chemicals are higher than those of HMX or RDX. It provides better impulse and detonation velocity when used in explosives and propellants. Along with better performance, this chemical fulfills high standards for munitions sensitivity [15]. However, the fate of CL-20 is little understood, and research into the possible adverse impacts of its accidental discharge into the ecosystem is required before producing this chemical on a wide scale [16].

In the military operation, RDX is a common ingredient in plastic-bonded explosives that are used to load a variety of ordnance. It is used in the civilian sector for items like fireworks and demolition blocks [17]. Due to its extensive use in various military and civilian operations worldwide during World War II, RDX has contaminated soil, sediments, and water [9]. The environmental release of RDX can result from military training operations, manufacturing facility effluents, unexploded munitions, and the dumping of explosive chemicals into the environment [18]. Approximately 12 mg/L of RDX may be released into the ecosystem as process wastewater during its synthesis [19]. HMX is frequently used as a raw element in propellant formulations, nuclear equipment, and munitions production due to its high detonation rate and chemical stability. It is among the most potent explosives produced due to its significant molecular weight. Furthermore, it is frequently employed as an essential part of secondary explosives [20,21]. Residual explosives and other compounds used in the production process are typically present in considerable amounts in the wastewater produced by explosive production facilities [2,22]. Moreover, it is anticipated that the manufacturing of CL-20 and its application in propellants and weapons can also contaminate the environment. Like RDX and HMX, it may contaminate the environment during production and utilization [23].

## 2. Physicochemical and Toxicological Properties

Nitramine compounds RDX, HMX, and CL-20 are heterocyclic molecules. The oligomers RDX and HMX are monocyclic nitramines, composed of the repeating structural unit CH_2_NNO_2_ (methylene nitrate). RDX and HMX have six and eight-membered rings. Both chemicals have a two-dimensional structure. HMX has four such units, whereas RDX has three. HMX is superior to RDX because it makes a larger ring structure [11]. There are four polymorphisms of HMX: orthorhombic (α), monoclinic (β), hypoclinic (γ), and hexagonal (δ). The β-form is recognized as the most stable phase due to its low impact sensitivity, stability, and maximum density [3]. On the other hand, CL-20, a polycyclic nitramine, has the repeating unit CH-N-NO_2_ with distinctive C-C bonds [24]. As CL-20 looks like two RDX rings connected at several carbon atoms, it is known as a three-dimensional caged molecule [15]. Common physico-chemical properties of RDX, HMX, and CL-20 are shown in Table 1. RDX is a colorless, polycrystalline, and extremely stable chemical. RDX has lower water solubility and can enter groundwater through soil profile because it limited adsorption [11]. Moreover, RDX has a poor soil adsorption coefficient, which increases the risk that it would contaminate groundwater close to military facilities, testing grounds, and conflict areas [25]. With a higher logK_W_ (1.92), CL-20 demonstrated a greater affinity for organic matter than both RDX (0.90) and HMX (0.16) [26]. Compared to RDX and HMX, CL-20 is expected to move through soil more slowly and cause less water pollution due to its low solubility and possibly strong binding to soil particles [27]. Since RDX, HMX, and CL-20 have relatively low water solubilities (40.0, 6.6, and 3.6 mg/L at 25 °C), they disperse differently in terrestrial and marine habitats [14,28,29].

The production process produces a lot of wastewaters that contains RDX and HMX explosive wastes. Many studies have reported the adverse effects of both explosives on plants, invertebrates, and animals, which have raised concerns about the accumulation of these hazardous and destructive explosives in the environment [12]. RDX is classified as a category C probable carcinogen [30,31]. RDX’s harmful effects have been studied in a number of model species. Researchers uncover the harmful effects of RDX on parental nematodes *Caenorhabditis elegans* (*C. elegans*) and its transgenerational reproductive toxicity, along with the signaling pathways implicated. RDX exposure up to 1000 ng/mL resulted in a marked rise in the generation of reactive oxygen species (ROS), death of germ cells, and a reduction in the number of eggs produced in *C. elegans* [32]. The median effective concentrations (EC_50_) for cocoon and juvenile production, 3.7 and 5.0 mg/kg, respectively, in freshly amended soil, showed that earthworm, *Eisenia fetida*, exposure to RDX had a negative impact on reproduction; however, the survival of adult earthworms was unaffected [33]. In northern bobwhites (*Colinus virginianus*), an avian species, data from dietary RDX exposure for 90 days showed a dose-dependent decline in total feed consumption, total egg production, and hen-housed production characteristics [34]. Moreover, adult zebrafish exposed to RDX over a long duration of time may be lethally poisoned at concentrations of 9.6 ppm. Furthermore, zebrafish exposed to sublethal quantities of RDX had little direct reproductive impact [35]. Moreover, Fathead minnows suffered from overt toxicity when exposed to RDX at the highest dose level, which led to 50% mortality and weight loss [36]. In three age groups of deer mice (*Peromyscus maniculatus*), the age-dependent toxicity of RDX or its nitroso metabolites was assessed at 21 days, 50 days, and 200 days. These results indicate that RDX is the most potent chemical examined, and all chemicals may be toxic with respect to age [37].

HMX is classified as a group D carcinogen. It has been discovered that HMX has harmful effects on microorganisms, aquatic organisms, and earthworms. Long-term exposure to explosive contaminants, such as RDX and HMX, significantly decreased fungal populations, enzyme activity, and microbial biomass. According to these results, soil microbes are susceptible to explosive contaminants [38]. A study evaluating HMX accumulation and its consequences in green anoles (*Anolis carolinensis*) has been reported. Eggs exposed to 2000 mg/kg HMX in the soil during incubation had a 50% lower hatching success rate, which was the only notable impact on the eggs. HMX exposure had no effect on hatchling growth or survival [39]. Research on HMX exposure revealed species-specific impacts in mammals (rabbit (*Oryctolagus cuniculus*)), amphibians (redbacked salamander (*Plethodon cinereus*)), and reptiles (Western fence lizard (*Sceloporus occidentalis*)), three vertebrate species. While salamanders showed no effects at ≤1970 mg/kg HMX, lizards died at high oral dosages at 5000 mg/kg HMX, most likely due to gastrointestinal impaction. With an LD_50_ of 93 mg/kg, rabbits showed neurological symptoms, which are more sensitive than those in amphibians and reptiles [40]. A concentration-dependent reduction in food intake, body mass, and egg production was observed in northern bobwhites (*Colinus virginianus*) exposed to HMX in their diet. Rather than being directly toxic, physiological modifications were associated with reduced food intake. Consequently, HMX is unlikely to pose significant dangers to wildlife in military areas, as it primarily causes feed aversion [41]. The earthworm (*Eisenia andrei*) has been used to evaluate the sublethal and long-term effects of HMX. The effects of HMX on cocoon production are evident in reproductive outcomes, including the quantity of juveniles and their biomass. Adult development generally does not correlate strongly with changes in reproduction ability, though [42,43]. Moreover, HMX has cytotoxic and mutagenic effects on the bacterial strain S. typhimurium TA98 in the absence of metabolic stimulation, whereas RDX has no carcinogenic potential [44].

Because of its structural resemblance to RDX and HMX, CL-20 is likely to have comparable harmful and possibly carcinogenic effects [23,45]. The toxicity and absorption of these chemicals in perennial ryegrass were evaluated. Ryegrass growth in different soils was unaffected by a 21-day exposure to RDX, HMX, or CL-20. HMX and RDX were detected in shoots; however, CL-20 was only detected in the roots. All three explosives can accumulate in plants, potentially lead to their accumulation in the food chain [10]. Communities of native nematodes and microarthropods responded differently to CL-20 in Sassafras sandy loam soil. Under CL-20, nematodes either remained the same or increased in quantity, but after 4-8 weeks, microarthropods drastically decreased. The abundance of microarthropods declined with increasing CL-20 concentrations, despite a partial recovery by 12 weeks [46]. Moreover, for the earthworm (*Eisenia andrei*), CL-20 is a reproductive toxin that can be toxic at higher doses. In soils with a high organic carbon content that facilitates CL-20 adsorption, its harmful effect can be reduced [47]. Additionally, the earthworm *Eisenia fetida* exhibits neurotoxicity when exposed to CL-20 through dermal contact [48,49]. Furthermore, CL-20 demonstrated substantial toxicity to the potworms *Enchytraeus albidus* and *Enchytraeus crypticus* at relatively low concentrations (0.08–0.7 mg/kg), dramatically decreasing adult survival and juvenile production. However, at far higher quantities, RDX and HMX exhibited no noticeable impact on survival, indicating that CL-20 is more Secondary pollution harmful [50]. CL-20 has also been shown to be toxic to avian species. CL-20 or its degradation products may produce adverse developmental effects in Japanese quail embryos (*Coturnix coturnix japonica*), although adults showed minimal effects [51]. However, freshwater green algae *Selenastrum capricornutum*, marine bacteria *Vibrio fischeri*, terrestrial higher plants, and microbes are all shown to be unaffected by CL-20 [9]. The toxic effects of these explosives have been summarized in Table 2.

It has also been found that these explosives can harm the liver and the central nervous system. Cough, headache, exhaustion, and nausea are additional harmful side effects [7,8]. RDX is considered a potential human carcinogen and can induce blood pressure disturbances, seizures, and reproductive problems. Changes in DNA methylation, oxidative stress, and mitochondrial dysfunction are all associated with its carcinogenicity [52]. Although HMX is not a known carcinogen, its intermediates and metabolites have mutagenic characteristics. It causes toxicity through oxidative stress, mitochondrial malfunction, and DNA alkylation. HMX could behave as a co-carcinogen by enhancing the effects of other genotoxic substances, even if its direct carcinogenic effects are unknown. The potential for mutation and cancer caused by CL-20 has not been covered literature [3,4,7,23,53]. Both aquatic and terrestrial species are poisoned by the residues and transformed metabolites they release into the environment. To mitigate the risk to the environment and public health, wastewater carrying these toxic compounds must be treated. A significant obstacle is the limited information available regarding the combined and chronic effects of explosives and their byproducts. Further toxicological investigation, site-specific risk assessments, and innovative approaches are therefore necessary for efficient management, cleanup, and long-term sustainability.

## 3. Industrial Production and Associated Wastewater

**Figure 1:**
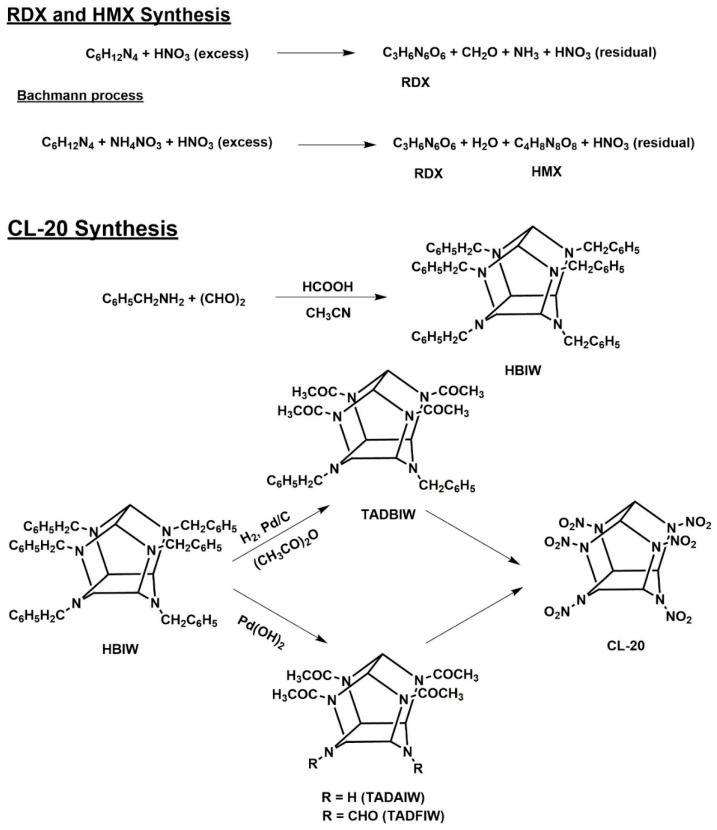
Synthesis of Nitramines RDX, HMX, and CL-20 [54,55,56,57].

Industry synthesizes RDX primarily by two methods. In direct nitration, hexamine reacts with nitric acid, yielding RDX with formaldehyde and ammonia as byproducts. The next method is the Bachmann procedure, which additionally uses nitric acid and hexamine along with acetic anhydride and ammonium nitrate (Figure 1). A second explosive product (HMX) is concurrently produced by this reaction [54,55]. The first step in the synthesis of CL-20 is the formation of the cage structure through the condensation of glyoxal with benzylamine, which yields hexabenzylhexaazaisowurtzitane (HBIW). HBIW is hydrogenated under reductive debenzylation using a palladium catalyst, which produces tetraacetyl derivatives (TADBIW: 4,10-dibenzyl-2,6,8,12-tetraacetyl-2,4,6,8,10,12-hexaazaisowurtzitane, TADAIW: 2,6,8,12-tetraacetyl-2,4,6,8,10,12-hexaazaisowurtzitane, TADFIW: 4,10-diformyl-2,6,8,12-tetraacetyl-2,4,6,8,10,12-hexaazaisowurtzitane). After that, nitrosonium (NOBF_4_) and nitronium salts (NO_2_BF_4_) are used to transform these intermediates into CL-20. Alternatively, CL-20 can also be produced by heating concentrated nitric acid (98%) to a high temperature. Nitric and sulfuric acid mixtures can be used to directly nitrate TADAIW and TADFIW (Figure 1) [56,57].

Large volumes of pollutants, including nitrate, 2,4,6-trinitrotoluene (TNT), RDX, HMX, nitroglycerine, etc., are found in wastewater from the explosives and ammunition manufacturing sectors. Additionally, the RDX and HMX manufacturing effluents contain a number of byproducts, including hexamine, high levels of nitrate, ammonium, and acetate in the wastewater generated to produce these explosives. The compositions of wastewater contaminated by these chemicals are shown in Table 3. Treating these explosives in an environmentally friendly way is essential to following guidelines and minimizing the negative environmental impact of RDX and HMX. However, because of their high chemical oxygen demand (COD) levels and extremely acidic character, HMX effluents present numerous treatment challenges [2,20,58]. Moreover, the main processes that produce wastewater containing CL-20 in industrial and military manufacturing are the nitration reaction and formulation procedures. This effluent is distinguished by its high levels of CL-20, nitric acid, sulfuric acid, and other organic solvents, as well as its excessive acidity [59,60]. Because explosive chemical exposure has a negative impact on both humans and the environment, these chemicals must be treated before their discharge.

## 4. Conventional Treatment Approaches

Developing an efficient and cost-effective treatment system is difficult because of the various compositions and very acidic pH, and very high COD values of the wastewater from explosive-producing facilities [61]. However, the demand for establishing effective and economically viable treatment technologies for explosive-contaminated wastewater and, as well as other water bodies, is increasing. Conventionally, a variety of treatments, including chemical, thermal, physical, and biological approaches, are used to treat wastewater contaminated by explosives. A variety of physical treatment techniques, such as sedimentation, filtration, and centrifugation separation, are typically used to manage explosive wastewater that contains insoluble components or colloidal matter. By separating and eliminating suspended particles and particulate matter from the water using physical processes, these methods increase clarity and lower the load of contaminants [7].

A variety of physical and chemical treatment techniques, including advanced oxidation processes (AOPs), alkaline hydrolysis, chemical reduction, and physical adsorption, have been studied for explosives remediation from groundwater and wastewater [63].

### 4.1. Adsorption

The adsorption method is widely used to remove harmful contaminants and is considered an attractive option due to its excellent cost-effectiveness and selectivity. Numerous researchers have documented activated carbon-based adsorption of harmful chemicals, which is commercially accessible. Hazardous compounds found in wastewater can be effectively removed using granular activated carbon (GAC). Granular activated carbon has been used in fixed-bed or fluidized-bed reactors to remediate wastewater containing nitramines from the explosives industry [7,20,64]. The effectiveness of granular activated carbon in removing RDX and HMX from polluted groundwater has also been investigated. For this investigation, several types of GACs were chosen, and Calgon F400 was the most successful in removing RDX and HMX [65]. Moreover, RDX wastewater has been treated photocatalytically using activated carbon fiber (ACF) cloth filled with nanoscale TiO_2_ particles. In 120 min, RDX degradation was achieved 95% [66].

Additionally, mesoporous silicas produced from oxidizing Ammonium Perchlorate were employed as adsorbents for HMX removal. The mesoporous silica SBA-15 treated with ammonium perchlorate-HNO_3_ demonstrated excellent HMX selectivity and adsorption capability [67]. Moreover, using N-isopropyl acrylamide and polyacrylic acid, hydrogels have been developed. These hydrogels showed the capacity to extract nitro explosives from aqueous solutions through batch absorption studies. The hydrogels showed stability and have potential for use in pollution removal from the environment [68].

### 4.2. Chemical Reduction

Zero-valent iron is widely utilized in groundwater treatment and has been documented as an effective reducing agent. As the explosives contain a reducible nitro group, nZVI can be an effective chemical treatment. The application of nano-zero-valent iron (nZVIs) has emerged as a very successful technique because of their large specific surface area [69]. The potential application of nZVIs to remediate wastewater contaminated by explosives has been examined in a study [70]. The substantial reduction efficiency and high reaction rate for RDX and HMX imply that nZVIs could be effective and appropriate materials for the degradation of explosives-contaminated wastewaters [70]. The notably promising reductant nZVI@MG (nZVI assembled on magnetic Fe_3_O_4_/reduced graphene oxides composite) demonstrated improved CL-20 elimination. The degradation of CL-20 by nZVI@MG was driven by an integrated adsorption-reduction mechanism, as follows pseudo-second-order kinetics approach. The study suggests that nZVI@MG is a viable alternative for CL-20 cleanup from contaminated wastewater, and degradation studies identified two pathways: denitrification and nitro reduction [71]. Another study evaluated the potential of nZVI for cleaning up explosives-contaminated soil and groundwater in a permeable reactive barrier (PRB) system, column trials, and batch testing. Explosives were reduced fast, with elimination efficiencies reaching up to 95% in batch experiments compared to column and PRB systems [72].

### 4.3. Advanced Oxidation Processes (AOPs)

Wastewater containing persistent organic contaminants is frequently cleaned using advanced oxidation processes (AOPs). It is considered to be most effective methods for treating explosive chemicals that have contaminated soils and water [73]. In order to oxidize resistant organic contaminants, the AOPs produce a substantial amount of hydroxyl radicals. Hydroxyl radicals can be produced by a number of processes, including Fenton (H_2_O_2_/FeSO_4_), photolysis (UV), photo-peroxidation (UV/H_2_O_2_), UV/O_3_/H_2_O_2_, photocatalysts (UV/TiO_2_), and O_3_/H_2_O_2_ [63]. AOPs that have been studied for HMX mitigation include photocatalysis employing different types of Fenton processes, photo-Fenton (UV/Fenton’s reagent), electro-assisted photo-Fenton procedures, etc. [20].

The degradation of RDX was examined using photo-peroxidation and photo-Fenton oxidation. The photo-Fenton technique was discovered to be more effective than the photo-peroxidation procedure. It was discovered that the degradation process followed first-order reaction kinetics. The TOC, TN, and COD values were observed to have decreased by 91.6%, 70.7%, and 93%, in the UV/Fenton process, and 85.1%, 69.1%, and 88.7%, respectively, in the UV/H_2_O_2_ treatment [74]. Moreover, explosive-polluted wastewater has been effectively treated using the Fenton process. The obtained oxidation efficiency was 75% and the COD elimination efficiency was 70%. The elimination effectiveness was improved by increasing the concentration of Fe^2+^ and the level of H_2_O_2_ [63]. Advanced oxidation approaches have been evaluated on a bench-scale study to treat groundwater from a US Army facility that was contaminated by explosives. Pollutants have been effectively eliminated by ozonated systems. UV irradiation used along with hydrogen peroxide and ozone provided the best performance, eliminating RDX and HMX entirely in ten minutes [75].

A study examined the treatment of RDX wastewater using direct photolysis, photo-peroxidation, and photo-Fenton oxidation. Direct photolysis with a 125 W medium-pressure lamp resulted in a 71% reduction, while photo-peroxidation further enhanced the degradation rate. The most efficient method was photo-Fenton oxidation, which removed 42% of the nitrate, 90% of the COD, and 98% of the RDX in a 125 W UV/Fenton system and 158% of the RDX in a 6 W UV-Fenton system. The rate of reaction for the breakdown of RDX by these three processes was pseudo-first order [18]. Moreover, when CL-20 was photolyzed in a Rayonet photoreactor at 300 nm in aqueous solution, it broke down quickly and formed nearly stoichiometric levels of nitrite and HCOOH [24]. In a study, nano-sized titanium dioxide (nano-TiO_2_) is used to photocatalytically remove RDX wastewater in a simulated sunlight environment. Using nano-TiO_2_ as the photocatalytic catalyst was shown to be highly effective in degrading RDX effluent. The RDX degradation caused by photocatalysis is either the same as or higher than Fenton oxidation [66]. The photocatalytic breakdown of RDX and HMX in a circular reactor with a UV lamp and TiO_2_ has been investigated. Significantly more degradation was accomplished by the combined UV-TiO_2_ system than by either UV or TiO_2_ separately. There was pseudo-first-order kinetics to the deterioration. This process proceeded according to pseudo-first-order kinetics [76]. Furthermore, the degradation of RDX and HMX from real wastewater was accomplished using the electro-assisted Fenton method. Compared to Fenton and photo-Fenton processes, the electro-assisted Fenton method produced a greater degree of degradation. The higher efficiency of the electro-assisted process was due to faster Fe^3+^ reduction and increased hydroxyl radical production [77].

Recalcitrant organic wastewater can be treated by electrochemical oxidation (ECO), which utilizes an anode and a cathode to convert electrical energy into chemical reactants. It can occur directly on the anode surface or indirectly through chemical mediators, such as ozone and hydroxyl radicals. By efficiently mineralizing contaminants and lowering toxicity and COD, EO is a viable technique for treating explosive wastewater. According to a study, the treatment of HMX wastewater can be accomplished using an electrochemical oxidation system with a Ti/PbO_2_ electrode. In 180 min, the maximum HMX elimination effectiveness of 81.2% was attained. In HMX wastewater, the two main stress types were oxidative and protein stress. After a 90 min breakdown period, the byproducts produced had comparatively greater molecular toxicity levels before eventually changing into less toxic or non-toxic end products [78]. Bio-electrochemical systems achieve higher removal rates of NACs compared to traditional biological treatment, with low energy demand compared to traditional electrochemical systems, and thus are suitable for nitramine treatment [79].

### 4.4. Alkaline Hydrolysis

Alkaline hydrolysis could be a cost-effective method for treating water contaminated with explosives. When RDX and HMX were hydrolyzed in an aqueous solution (pH 10–12.3), the degradation of these chemicals was followed by the formation of ring cleavage products and nitrite. CL-20 can produce HCOOH, NH_3_, and N_2_O as a result of denitration [80]. The kinetics of RDX and HMX hydrolysis were examined at temperatures ranging from 50 to 80 °C and pH values between 10 and 12. The hydrolysis rate of HMX is significantly slower than that of RDX, and it may become rate-limiting when treating RDX and HMX mixture [81].

### 4.5. Limitations of Physical and Chemical Methods

As mentioned above, the removal of explosives from contaminated soil and water also involved the employment of a variety of physical and chemical methods, including adsorption, chemical reduction, and advanced oxidation (Table 4). These techniques are typically costly and result in just separation or partial transformation rather than complete compound degradation [11]. Furthermore, the advanced oxidation process has certain drawbacks, including the production of unwanted products, the requirement for additional treatment, the increased cost of the catalyst, and the chemicals needed for the Fenton and photo-Fenton processes [20]. Chemical reduction using zero-valent iron particles may facilitate transformation and detoxification, but it is not a comprehensive remediation technique on its own. Additionally, resistant and harmful reaction contaminants may be generated during the chemical reduction processes [82]. The adsorption process is commonly used to remove nitro-explosives from contaminated waters. The adsorption process has certain drawbacks, such as the use of chemicals for adsorbent reuse, treatment of wasted adsorbent, and further processing of the regenerated mixture that contains explosive remains. The primary drawback of physical and chemical approaches is their limited applicability to ex situ situations [20,63,83,84]. The need for an environmentally friendly method to mitigate the harmful effects of these chemicals has thus arisen, and bioremediation has emerged as a potential treatment solution.

## 5. Biodegradation Potential and Mechanisms

Numerous substances, including xenobiotics, may have been degraded by microorganisms [42]. Cyclic nitramines, which do not possess aromatic stability, can be biodegraded by a variety of enzymatic processes, such as ring cleavage at N-N or C-N bonds, hydride transfer, hydroxylation, reduction to nitroso or hydroxylamino derivatives, or nitro group removal). The ring is destabilized by such processes, which produce various intermediates that can break down spontaneously in water. In aqueous conditions, several unstable secondary intermediates break down abiotically. Therefore, a combination of biotic and abiotic processes is involved in their degradation. These processes produce common byproducts, such as formic acid and glyoxal from CL-20 degradation, or more toxic nitroso, hydroxylamino, and amino derivatives, and less toxic nitrite, nitrous oxide, ammonia, and formaldehyde from RDX and HMX degradation [8].

### 5.1. Biodegradation of RDX

**Figure 2:**
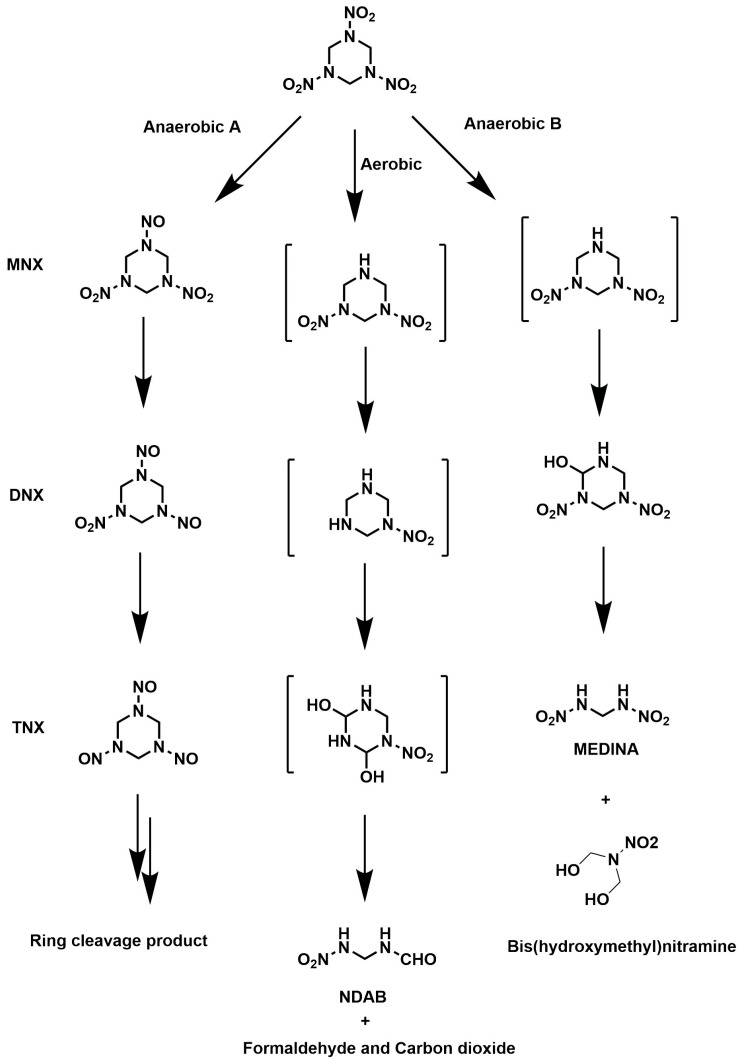
RDX biodegradation pathways under aerobic and anaerobic conditions [3,9,11,85,86,87].

Numerous bacteria have been discovered to biodegrade RDX in both aerobic and anaerobic environments. Since RDX is a significantly oxidized molecule, ring breakage could result from an initial microbial denitration that weakened the inner C-N bonds [84]. Two routes for the degradation of RDX under anaerobic conditions have been documented: one was the reduction of RDX’s nitro groups to nitroso derivatives, and the other was a direct ring cleavage that produced methylene dinitramine (MEDINA) and bis(hydroxymethyl)nitramine (BHNA) with or without prior denitration (Figure 2). The reductive intermediates such as hexahydro-1-nitroso-3,5-dinitro-1,3,5-triazine (MNX), hexahydro-1,3-dinitroso-nitro-1,3,5-triazine (DNX), and hexahydro-1,3,5-trinitroso-1,3,5-triazine (TNX) are formed by successive reductions of nitro groups of RDX by the two-electron reductive route (Figure 2) [3,9,11]. It was predicted that reducing these compounds further to hypothetical hydroxylamino derivatives would destabilize the ring, causing hydrolytic breakdown and resulting production of ring cleavage products such as formaldehyde, methanol, and hydrazines. Following two single-electron transfer pathways, the RDX degradation process aerobically produces 4-nitro-2,4-diazabutanal (NDAB) by removing two nitrite groups and then cleaving the ring (Figure 2) [85,86,87]. Nitroso intermediates were not found in studies conducted aerobically, indicating that the degradation processes in anaerobic and aerobic environments are distinctive.

An aerobic bacterium known as *Gordonia* sp. has been isolated from the contaminated soil and was found to degrade RDX aerobically, producing 4-nitro-2,4-diazabutanal [88]. The potential of *Janibacter cremeus* to degrade RDX in aqueous medium was assessed under aerobic conditions. This investigation demonstrated that *J. cremeus* was a promising degrader. In *J. cremeus*, nitroreductase played a crucial role in the RDX degradation pathway. The denitration process, which produces BHNA and MDNA, may be a key degradation pathway in RDX degradation because of the significant nitrite release [89]. *Shewanella oneidensis* MR-1 can effectively degrade RDX by co-metabolizing without utilizing it as a carbon, nitrogen, or energy source in anaerobic conditions. Two degradation pathways were followed by the bacterium: a minor one that involved ring cleavage via the denitration process, and a significant one that involved stepwise nitro reduction. The study also found that the c-type cytochrome *cymA* gene plays a role in RDX transformation [90]. In another study, *Shewanella putrefaciens* CN32, which exhibited less activity either by itself or in combination with lepidocrocite, was able to degrade RDX more efficiently when riboflavin was present. In 78 h, CN32 totally reduced 110 µM RDX in the presence of riboflavin in anaerobic conditions. Ring cleavage of RDX was indicated by the observed transformation products, which included ammonium, formaldehyde, and nitroso intermediates [91].

*Clostridium bifermentans* strain HAW-1 is a rapidly RDX-degrading, essentially anaerobic bacterium that utilizes RDX as its sole source of carbon and nitrogen. Strain HAW-1 degrades RDX by first reducing it to MNX, then denitrating and decomposing it. Although strain HAW-1 had a high rate of RDX removal, mineralization was just 3% [92]. In another study, *Clostridium* sp. strain EDB2, an anaerobic strain, was discovered from a maritime sediment. Through N-denitration, strain EDB2 can degrade RDX while simultaneously releasing nitrite. Additionally, the study verified that the primary cause of the chemotaxis response in strain EDB2 was the nitrite produced from the explosive degradation [14]. A xenobiotic is often metabolized more quickly by the mixed cultures than by the isolated strains. *Gordonia* sp. KTR9, an aerobic strain, produces NDAB as a byproduct of RDX degradation, which it utilizes as a nitrogen source. As the only source of nitrogen, NDAB is degraded by *Methylobacterium* sp. strain JS178 in aerobic conditions. Complete degradation of RDX was achieved using the combination of strains KTR9 and JS178. The rate at which KTR9 alone or in co-culture with JS178 degraded RDX was the same [93].

Some *Rhodococcus* strains (Strains DN22, 11Y, and A) showed slow degradation of RDX in microaerophilic environments but did not degrade it under strict anaerobic conditions. The rate of degradation was around 60 times slower in microaerophilic conditions as compared to aerobic conditions. These strains utilize RDX and succinate as a sole source of nitrogen and carbon. RDX metabolite, such as MEDINA, was found only in aerobic conditions, but NDAB was found in both microaerophilic and aerobic conditions [94]. Some *Rhodococcus* strains, YH1, T7, and T9N, that were recovered from polluted sites in Israel, can also degrade RDX and utilize it as a nitrogen source as glucose as a carbon source. It was discovered that the cytochrome enzyme in these strains accelerated the denitration pathway during the RDX degradation process under aerobic conditions. The cytochrome P450 is encoded by a highly conserved gene *xplA*, and the activity of this enzyme has been determined experimentally [86,95,96]. Furthermore, the effect of numerous nitrogen-containing chemicals, including TNT, ammonium, nitrite, and nitrate, has been investigated in RDX degradation. The RDX degradation rate was found to be significantly affected by both organic and inorganic nitrogen sources. Ammonium completely prevented the process of RDX degradation by strain YH1, whereas nitrate and nitrite delayed it [97]. Research has demonstrated that TNT inhibits the degradation of RDX by strains YH1, T7, and YY1. Moreover, the impact of perchlorate and chlorate has also been investigated in the aerobic degradation of RDX. *Rhodococcus* strains YH1 and T7 and *Gordonia* YY1 showed varying reactions to the co-contaminants, with strain T7 being most impacted and strain YH1 being the least. Water contaminated by RDX and containing a variety of co-contaminants that are environmentally significant can be bioremediated using all three strains [30,98].

### 5.2. Biodegradation of HMX

**Figure 3:**
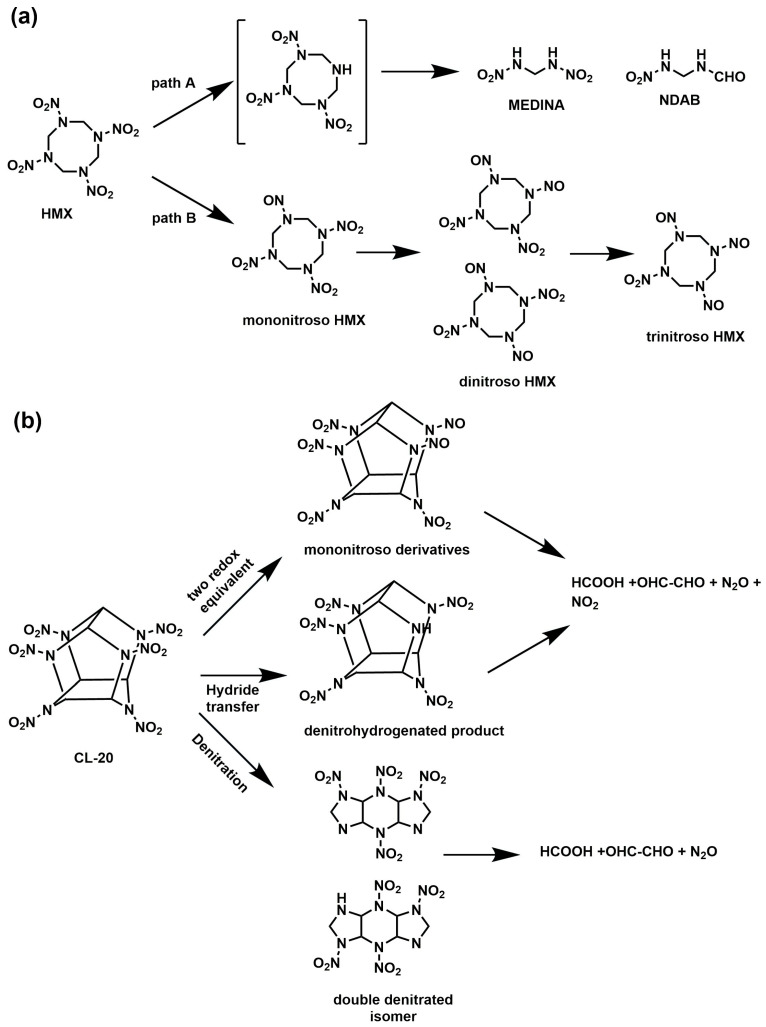
HMX and CL-20 biodegradation pathways. (**a**) HMX biodegradation pathway, and (**b**) CL20 biodegradation pathway [3,9].

The monomeric units of CH_2_-N-NO_2_ in the HMX and RDX are structurally identical. The mechanism of HMX biodegradation is comparable to that of RDX, despite HMX being demonstrated to be more resistant to biodegradation than RDX. The majority of research on the anaerobic process of HMX degradation identifies nitroso derivatives as intermediates that are produced when the nitro groups undergo a two-electron reduction. Octahydro-1-nitroso-3,5,7-trinitro-1,3,5,7-tetrazocine (mononitroso-HMX) is the most common biproduct; however, octahydro-1,3-dinitroso-5,7-dinitro-1,3,5,7-tetrazocine or octahydro-1,5-dinitroso-3,7-dinitro-1,3,5,7-tetrazocine (dinitroso-HMX) are also reported, and octahydro-1,3,5-trinitroso-7-nitro-1,3,5,7-tetra-zocine (trinitroso-HMX) is also found [8]. Another mechanism of HMX biodegradation involves ring cleavage and forms intermediates MEDINA and BHNA (Figure 3a) [3,9]. HMX can be metabolized by several H_2_-releasing fermentative anaerobic bacteria. Among them, *Clostridium bifermentans* strain HAW-1 transformed HMX, identifying the two important intermediates, MEDINA and the mononitroso derivatives. The findings indicate that HMX degradation required two different kinds of enzymes: one for transformation to the nitroso compounds (nitro reductase) and another for denitration (hydrogenase) [99].

A study demonstrated that *Bacillus aryabhattai* can effectively metabolize HMX and has a high resistance to it. HMX is metabolized extracellularly, and the resulting byproducts then transform into intracellular purines, amino sugars, and nucleoside sugars that subsequently take part in cell metabolism [21]. Another study investigated HMX degradation in aerobic conditions using the *Planomicrobium flavidum* strain S5-TSA-19, which was isolated from soil contaminated with explosives. Over the course of 20 days, the strain S5-TSA-19 degraded 70% of HMX, releasing nitrite and byproducts such as MEDINA and N-methyl-N,N′-dinitromethanediamine [100]. Additionally, the transformation of HMX catalyzed by the enzyme xanthine oxidase (XO) has been studied. Both aerobic and anaerobic biotransformation rates showed that the reaction was higher in anaerobic conditions. The products formed during the reaction included nitrite, MEDINA, NDAB, etc. [101]. Using denitrifying bacterial isolates, the biodegradation of high-explosive manufacturing effluent, including RDX and HMX, has been studied. Two *Bacillus* strains (HPB2, HPB3) and *Pseudomonas* (HPB1) were used to biodegrade the effluent. The results indicated that HMX and RDX underwent biodegradation under denitrifying conditions. It was discovered that the isolated HPB1 was an effective strain for HMX degradation. In contrast to the HPB2, the HPB1 was observed to have a lower degradation efficiency for RDX [102].

### 5.3. Biodegradation of CL-20

For CL-20, three distinct biotransformation routes have been proposed. In the first pathway, a nitro group is released through denitration. The second pathway produces the denitrohydrogenated intermediates through a hydride ion transfer. The third pathway utilizes two redox equivalents to reduce CL-20 to its mononitroso derivative (Figure 3b) [9]. Another work was reported to isolate a single strain of bacteria from the activated sludge that is capable of degrading CL-20. Within 48 h, the microbial community (isolated from activated sludge) and *Pseudomonas* sp. ZyL-01 caused the CL-20 concentrations to be reduced by 75.21 μg/mL and 74.02 μg/mL from the 100 µg/mL initial values. Additionally, after 14 days of incubation, ZyL-01 was able to break down 98% CL-20 of the actual effluent in the presence of glucose [103]. *Escherichia coli* nitroreductase was discovered to catalyze a one-electron transfer to CL-20, which subsequently followed the denitration pathway. Under anaerobic and aerobic conditions, CL-20 biotransformation rates were 3.4 ± 0.2 and 0.25 ± 0.01, respectively [104]. Another study used two enzymes, a diaphorase from *Clostridium kluyveri* and a dehydrogenase from *Clostridium* sp. EDB2, to investigate the hydride transfer to CL-20. Using NAD(P)H as a hydride source, these enzymes biotransformed CL-20 and generated N-denitrohydrogenated products. Both enzymes demonstrated stereospecificity for the pro-R hydrogen of NAD(P)H The summary of nitramine-degrading microorganisms, pathways, and degradation products is compiled in Table 5.

### 5.4. Biodegradation of RDX, HMX, and CL-20 by Fungi

The widespread occurrence of fungi in nature and their ability to produce a wide variety of extracellular enzymes make them potentially useful in the degradation of nitramines. Fungi are capable of breaking down chemicals by using a variety of enzymes [11]. Researchers isolated four new aerobic RDX-degrading fungi, HAW-OCF1, HAW-OCF2, HAW-OCF3, and HAW-OCF5, from a coastal area on Oahu Island, Hawaii. These fungi have been provisionally identified as associated with the *Rhodotorula*, *Bullera*, *Acremonium*, and *Penicillium* genera, respectively. In 58 days, these isolates metabolized 15–34% of RDX, as indicated by CO_2_ released. The results of this study suggested that RDX broke down through at least two main initial pathways: ring cleavage to MEDINA and reduction to MNX before ring cleavage [106]. *Phanerochaete chrysosporium* is used in another study to describe the aerobic degradation of HMX. This strain can degrade HMX in liquid culture. Mono-nitroso derivatives were formed simultaneously with the degradation of HMX. The nitroso derivatives were further degraded through two possible pathways: α-hydroxylation before ring cleavage and N-denitration [107]. CL-20 can be effectively broken down by the white-rot fungus *Phanerochaete chrysosporium* and *Irpex lacteus* in an aerobic environment. The production of ^14^CO_2_ from [^14^C]-CL-20 indicated that *P. chrysosporium* was able to mineralize CL-20 efficiently. An initial denitration process occurred on CL-20 before ring cleavage was shown by the utilization of pure manganese peroxidase [108].

## 6. Biological Explosives Contaminated Wastewater Treatment Technologies

Numerous aerobic and anaerobic microbes can be employed in biological wastewater treatment to mitigate various types of contaminants found in water. Numerous anaerobic and aerobic microbes can be employed in biological wastewater treatment to lower the biochemical oxygen demand of water and various contaminants. In water treatment facilities, activated sludge is essential as an inoculum to start microbial processes in biological wastewater treatment. Many methods are utilized to remove pollutants, including membrane bioreactors, sequencing batch reactors, biofilm-based reactors, upflow anaerobic sludge blanket, and traditional activated sludge [109,110].

### 6.1. Activated Sludge/Aerobic Granular Sludge Process

The most common and extensively used biological wastewater treatment method worldwide is the activated sludge process. The primary objective of this procedure is to remove the biodegradable and soluble substances from wastewater in a reactor [111]. However, nitramine CL-20 did not undergo significant biodegradation in activated sludge; instead, hydrolysis, rather than microbial activity, was most likely the cause of the reported degradation. Nevertheless, the hydrolysis products of CL-20 were successfully mineralized into carbon dioxide. Therefore, it is possible to remove CL-20 and its byproducts from contaminated water by combining alkaline hydrolysis with aerobic microbial treatment [112]. Another study demonstrated that over 95% of toxic insensitive munition chemicals, including 2,4-dinitroanisole (DNAN) and 3-nitro-1,2,4-triazole-5-one (NTO), were successfully broken down by a lab-scale aerobic granular sludge sequencing batch bioreactor. The average removal of RDX was 38.4%, indicating a lesser yet considerable degradation [113]. Moreover, using aerobic granular sludge cultured in synthetic wastewater containing sodium acetate and glucose, HMX-production effluent has been efficiently treated [58].

Sequencing batch reactors, or SBRs, are widely utilized for treating wastewater that is continuously contaminated with complex contaminants due to their advanced process control capabilities and operational flexibility. Wastewater from HMX manufacturing processes was treated using aerobic granules in SBR [114]. Throughout the process, the granules demonstrated significant biological activity, a high biomass retention rate, and great settling performance. After operating steadily for 40 days, aerobic granular sludge was highly successful in removing carbon and nitrogen compounds from HMX wastewater. Nitrogen removal efficiency of up to 80% and organic matter removal rates of up to 97.57% were achieved throughout the procedure [58]. The removal of high explosives RDX and HMX was evaluated over a 64-day period using SBR. The culture eliminated 95% of the RDX when treated with mixed carbon (glucose, glycerol, and succinate) and 68% of the RDX when given ethanol throughout each 4-day SBR cycle. The addition of ammonium nitrate enhanced the reduction of RDX in SBRs [115].

RDX has been examined for its sequential anaerobic–aerobic biodegradation. Complete degradation occurred in cultures cultivated under sequential anaerobic-aerobic conditions in 20 days, utilizing a consortium of bacteria from the activated sludge. Under aerobic conditions, no degradation was observed in the experiments. Complete degradation of RDX was also accomplished with *Bacillus pumilus* in 2 days, when compared to studies utilizing the bacterial consortium [116]. The decomposition of RDX can be inhibited by inorganic nitrogen molecules such as NOx (nitrate or nitrite), since bacteria prefer simpler nitrogen sources. By removing NOx and organics by anaerobic denitrification, nitrogen-deficient conditions were established for the aerobic degradation of RDX. Two sequencing batch reactor systems were used in this study: a single reactor with anaerobic and aerobic stages in its operation cycle, and a two-stage anaerobic-aerobic system. Both successfully eliminated NOx and completely biodegraded RDX aerobically under ideal conditions [96].

### 6.2. Membrane Bioreactor

An additional study established and improved a dual anaerobic–aerobic membrane bioreactor (MBR) system for reducing wastewater that contains HMX and RDX. Both explosives were successfully treated by the combination method. Complete treatment of RDX and HMX was achieved by the integrated system, with the aerobic system polishing and the anaerobic MBR performing the majority of the degradation. In the effluent, no typical RDX or HMX metabolites were found [117]. Moreover, to treat synthetic wastewater containing alkaline hydrolysis byproducts of RDX, a bench-scale anoxic membrane bioreactor (MBR) has been assessed. The wastewater had the same composition as hydrolysis wastewater, with nitrite and nitrate serving as electron acceptors and formaldehyde, formate, and acetate serving as carbon sources. In the MBR, almost 97.0% of % COD, 93.3% of % nitrate, and 55% nitrite were reduced [118]. RDX-containing wastewater was treated utilizing a dual anaerobic-aerobic membrane bioreactor system. The anaerobic MBR totally broke down the RDX in the wastewater, and no reduced byproducts were produced. During several operating phases, the dual MBR system was given a wider range of explosive ingredients, such as TNT, HMX, nitroguanidine, perchlorate, and nitrate, and it was found to biodegrade these substances [119].

### 6.3. Up-Flow Anaerobic Sludge Blanket and Modified Reactors

Anaerobic conditions are used in the biological wastewater treatment system known as the up-flow anaerobic sludge bed (UASB) reactor. It produces biogas, mostly methane, and enables the breakdown of organic compounds. Wastewater in this reactor passes through a bed of granular sludge, which acts as a biocatalyst to break down organic contaminants [120]. RDX could be effectively removed from an aqueous solution using anaerobic granular sludge from a mesophilic UASB reactor. The primary process that removed RDX was biodegradation. The RDX degradation process was not substantially facilitated by ammonium. Anaerobic granular sludge was found to degrade RDX more readily when glucose was present, whereas sulfate and nitrate showed the opposite effect [121]. In another study, anaerobic granular sludge from a mesophilic UASB reactor operated under nitrate- and sulfate-enriched conditions was used to evaluate the transformation of RDX and HMX. While nitrate was present, the transformation process was hindered by high nitrate levels. It was more beneficial to remove both chemicals via sulfate enrichment. In both cases, adding glucose improved the elimination of RDX and HMX significantly [122].

Moreover, it has been demonstrated that biological degradation in anaerobic fluidized bed reactors (AFBR) is a successful technique for eliminating RDX and perchlorate from contaminated effluent. The efficacy of this technique in treating the wastewater was evaluated using three AFBRs. The experimental findings showed that bioreactors containing one particular pollutant performed better than those containing both contaminants in terms of the biodegradation of RDX and perchlorate [123]. Another study examined the anaerobic degradation of groundwater pollutants trichloroethene (TCE) and RDX using two microbial cultures (anaerobic sludge and a facultative enrichment culture). Within 4–7 days, both cultures efficiently broke down RDX and its byproducts to levels below detection. TCE degradation was moderate at first but improved with acclimatization [124].

### 6.4. Biofilm-Based Reactors

Since biofilm-mediated systems offer substantial benefits over traditional techniques, this is indeed the most effective approach for treating industrial wastewater. In the fixed film bioreactor (FFBR), another study demonstrates the ability of the yeast *Pichia sydowiorum* MCM Y-3 for the treatment of HMX wastewater. Brick fragments were used as strong supports in an FFBR to help the *P. sydowiorum* culture’s biofilm growth and treat wastewater. The FFBR was executed in both continuous and batch modes. Nitrate was reduced by 50–55%, acetate by 70–88%, COD by 50–66%, and HMX by 28–50% [62]. Moreover, for the degradation of TNT, RDX, and pentaerythritol tetranitrate (PETN) in membrane catalyst-film reactors, another study assessed Pd^0^ nano-catalysts on H_2_-transfer membranes. In addition to nitro-reduction of TNT and denitration of RDX and PETN, it demonstrated remarkable catalytic activity. When wastewater was treated continuously, all three chemicals were removed to higher than 96% [125].

### 6.5. Phytoremediation and Constructed Wetlands

Using green plants, phytoremediation eliminates both organic and inorganic contaminants from water and soil. It is an economical and environmentally friendly technique that is frequently used to clean up the environment, including explosive pollutants. In order to degrade contaminants through oxidation and reduction reactions, plant enzymes are important [126]. *Methylobacterium* sp. strain BJ001, a symbiotic bacterium, was isolated from the hybrid poplars (*Populus deltoides* × *nigra* DN34). Furthermore, it was demonstrated that the isolated methylotroph could transform RDX and HMX in 40 days. MEDINA and a mononitroso RDX derivative were among the byproducts found from RDX transformation. In hydroponic conditions, researchers investigated the absorption and distribution of TNT, RDX, and HMX by hybrid poplars. TNT had been removed from the solution more quickly than HMX and RDX. The majority of TNT was absorbed in the root tissue, whereas RDX and HMX were absorbed in the leaves [127]. Constructed wetlands (CWs) may reduce wastewater contaminants in a combined way by utilizing the physical, chemical, and biological remediation processes created by plants, substrates, and microorganisms. Because it is inexpensive, easy to build, and very effective, CW is a promising technology that can be used to treat industrial wastewater [79]. According to a two-year study, RDX was successfully extracted from water at different loading rates using down-flow constructed wetlands, with the best results obtained during a 2-day retention time. In both nitrate and sulfate-rich zones, RDX degraded, and the removal of plants had no impact on RDX removal. RDX loading was proportional to the appearance of byproducts such as MNX, DNX, and TNX, and RDX levels in plant tissue were connected with the rhizosphere [128].

## 7. Integrated Treatment Using Physical and Chemical Methods

Conventional physical, chemical, and biological methods have been used to treat explosive-laden wastewater; however, they mainly transfer contaminants between phases without achieving complete removal. Biological degradation faces additional limitations, including slow processing, incomplete mineralization, generation of biological sludge, and sensitivity to pollutant concentration, pH, and temperature [20]. Complete mineralization of the pollutant is the main objective of any treatment process. This can be accomplished by combining the appropriate treatment techniques. Explosive wastewater treatment has achieved efficiency using a combination of biological and physical/chemical techniques (Table 6) [7].

### 7.1. Adsorption with Biological Treatment

Explosives are successfully removed from munitions wastewater using an integrated treatment that combines aerobic/anaerobic biodegradation with adsorption. The porous substrates promote the formation of biofilms and absorb pollutants for microbial action. The effective fixed-film bioreactors known as fluidized bed reactors (FBRs) employ bacteria that have been immobilized on fluidized media particles. They facilitate the growth of microbial activity. Moreover, they could provide the ideal environment for these potent chemicals to degrade. Granulated activated carbon (GAC)-based fluidized bed reactors (FBRs) for treating perchlorate and RDX-contaminated groundwater were assessed in a bench-scale study. Molasses and acetic acid were evaluated as electron donors to facilitate the concurrent reduction of both contaminants. Because of the sorption of activated carbon media, RDX’s breakthrough was low. In contrast, molasses proved less efficient in the acetic acid-fed FBR, where above 99.5% of RDX was biodegraded as indicated by mass balance calculations [64].

### 7.2. Ultrafiltration with Biological Treatment

The treatment of explosive effluent was studied using a bench-scale continuous flow anoxic membrane bioreactor equipped with a ceramic ultrafiltration membrane. The carbon sources were the byproducts of RDX hydrolysis, while the electron acceptors were nitrate and nitrite. Within 60 days of operation, high removal efficiency was attained: 93% nitrate, 55% nitrite, and 97% COD. The study showed that treating RDX hydrolysate-rich wastewater with a combination of biological treatment and ultrafiltration is a successful method [118].

### 7.3. Advanced Oxidation Integrated with Biological Treatment

The hydroxyl radicals produced by advanced oxidation processes (AOPs) like UV/H2O_2_ and electrochemical oxidation break down organic contaminants quickly. While biological treatments are environmentally beneficial, they take time to adjust; advanced oxidation methods quickly break down resistant substances. Their combination has shown significant efficacy in treating munitions effluent, especially when combined with oxidation as a pre-treatment. A study focused on the combined electrocatalytic reaction and anoxic–oxic (A/O) activated sludge technique for treating RDX wastewater. The pretreatment technique used an electrochemical device with a TiO_2_-NTs/SnO_2_-Sb anode for removing RDX from the effluent. The electrocatalytic technique significantly improved biodegradability by removing 39.2% of COD and 97.5% of RDX. By reducing toxicity and enhancing biodegradability, photocatalytic pretreatment can accelerate biological treatment periods. Nevertheless, Yang et al.’s investigation found that the accumulation of nitrophenols (NP) from the photolysis pretreatment of nitrobenzene (NB) wastewater reduced microbial activity, ultimately decreasing the overall biodegradation efficiency of NB in biological treatment systems. In contrast, combined UV photolysis and biodegradation increase efficiency by immediately eliminating NPs [79,129]. In summary, this combination approach was effectively used, and the electrochemical pretreatment successfully eliminated the resistant and hazardous organic contaminants [130].

### 7.4. Zero Valent Iron (ZVI) Integrated with Biological Treatment

The effectiveness of a combined biological and zero valent iron (ZVI) technique in eliminating munitions chemicals in a step-by-step treatment procedure was evaluated. A growing number of recent studies are evaluating the use of biological adsorption systems combined with nanoscale zero valent iron for the treatment of munitions in the water. The potential advantages of a combined ZVI and microbial system for treating RDX-contaminated water were assessed using soil microcosms. Compared to individual treatments, RDX was mineralized more quickly and thoroughly in microcosms modified with both ZVI and anaerobic sludge. The nitroso byproducts MNX, DNX, and TNX have been identified in each microcosm [131]. Another study showed that RDX was quickly reduced in flow-through columns and in microcosms added with ZVI powder. Anaerobic bacteria that consume cathodic hydrogen (from ZVI) increased the magnitude of RDX transformation. RDX’s toxicity to microorganisms was further decreased by reducing it with ZVI, which also improved its future degradation in aerobic or anaerobic settings. Thus, a sequential or mixed ZVI-biological treatment strategy may increase treatment effectiveness [132]. Moreover, ZVI pretreatment has been studied in another investigation to improve the biodegradability of RDX. The findings of both batch and column studies demonstrated that ZVI removed RDX quickly and completely. In a mixed culture, formaldehyde, a reduction byproduct of RDX, was easily mineralized [133].

### 7.5. Alkaline Hydrolysis Integrated with Biological Treatment

In a laboratory-scale investigation, the products of the alkaline hydrolysis of RDX and HMX (acetate, formate, formaldehyde, and nitrite) were treated in a denitrifying (anoxic) packed-bed upflow reactor. The findings demonstrated the effective degradation of alkaline hydrolysis products by biological nitrification as a subsequent treatment step. A 2-year investigation found that a reactor removed over 90% of the organic chemicals and nitrite [134]. Moreover, the initial hydrolysis products of CL-20 were substantially mineralized to carbon dioxide by activated sludge. The removal of CL-20 and its byproducts from wastewater and groundwater can be accomplished effectively by combining alkaline hydrolysis with aerobic biological treatment [112]. Altogether, a variety of biological systems can be utilized for nitramine wastewater treatment under different redox conditions (Table 6).

**Table 6:** Bioreactors for nitramine removal.

Treatment Methods	Explosives	Condition	References
Activated sludge	CL-20 hydrolysates	aerobic	[112]
Membrane Bioreactor	RDX, HMX	sequential anaerobic–aerobic	[117]
	RDX hydrolysates	anoxic	[118]
Sequencing Batch Reactors	HMX	aerobic	[58]
	RDX, HMX	oxygen-depleted conditions	[115]
Up-flow Anaerobic Sludge Blanket and modified reactors	RDX	anaerobic	[121]
	RDX, HMX	anaerobic	[122]
	RDX	anaerobic	[123]
Adsorption with biological treatment: Granulated activated carbon (GAC)-based fluidized bed reactors	RDX	anoxic	[64]
Ultrafiltration with biological treatment	RDX hydrolysate	anoxic	[118]
Advanced oxidation integrated with biological treatment	RDX	anoxic–oxic	[130]
Zero valent iron (ZVI) integrated with biological treatment	RDX	anaerobic	[131]
	RDX	aerobic or anaerobic	[132]
Alkaline hydrolysis integrated with biological treatment	CL-20 hydrolysates	aerobic	[112]
Sequential biological treatment	RDX	sequential anaerobic–aerobic	[116]

The availability of nutrients, co-metabolic support, and redox conditions all have a significant impact on the biodegradation efficiency of nitramine explosives in wastewater. In reducing (anaerobic) conditions, RDX and HMX are more efficiently broken down by microorganisms like sulfate-reducing bacteria and anaerobic granular sludge. This is especially relevant when electron donors like glucose, acetate, or ethanol are added, as these substances promote explosive degradation [122]. An investigation was conducted to examine the effects of various carbon substrates on the anaerobic degradation of RDX and HMX. Acetate and glucose considerably accelerated degradation in contrast to soluble starch and ethanol. The biotransformation was shown to be mostly dependent on the concentration of the carbon source [135]. Moreover, anaerobic treatment was performed on RDX and HMX effluent using a variety of co-substrates. Biodegradation was significantly accelerated by dextrose and acetate, although ammonium chloride and sodium sulfate had minimal impact. As the concentration of sodium nitrate increased, RDX and HMX’s breakdown efficiency decreased [136]. *Agrobacterium* sp. JS71 has also been shown to degrade CL-20 in soil microcosms by employing succinate as a co-substrate, highlighting the significance of microbial adaptability and carbon availability in the metabolism of explosives [23].

## 8. Future Perspectives and Conclusions

Globally, the increased use of nitramine explosives has led to serious contamination of both soil and water. The industrial production of these chemicals in propellants and wastewater from explosive production facilities is currently the primary cause of pollution. These chemicals have a negative impact on human health and ecosystems [9]. These chemicals cannot be adequately treated by traditional wastewater treatment methods. There are some drawbacks when using physical and chemical methods, such as increased energy use, higher remaining solids, expensive disposal, etc. Furthermore, different types of wastewaters cannot be adequately treated by a single technique [84]. Most of the drawbacks of physical and chemical procedures are addressed by biological methods. Biological methods involve employing microorganisms to degrade or remove these toxic chemicals. Another sustainable method that has several benefits over conventional methods is phytoremediation. By applying phytoremediation, explosives can be reduced. However, it takes longer for plants to grow, and plants can accumulate explosives, causing them to persist in the surroundings. These traditional treatment techniques that operate solely on physical, chemical, and biological processes are ineffective for completely removing these contaminants. Our review indicates that the combination of abiotic treatment with biological biodegradation is the most promising solution for achieving high-quality effluents from nitramine explosives-polluted wastewater. Combining two or more treatment methods is the best way to provide an effective treatment. However, the nature and composition of effluent may influence the establishment of a specific combined wastewater treatment method. Another important factor is the financial impact of the established combined technology. The world needs an integrated wastewater management method that is affordable, highly effective, and widely applicable to close the gap between the availability and need for clean water.

Microorganisms utilize these chemicals as a source of nitrogen, but it has a number of drawbacks, such as slow rates of degradation, sensitivity to harmful substances, their inability to effectively treat persistent pollutants, and their dependence on microbial communities that might not be beneficial for specific contaminants [8,136]. For example, genome shuffling of *Stenotrophomonas maltophilia* OK-5 improved the degradation of RDX [137]. These obstacles are being addressed by recent developments in metagenomics, genetic engineering, and omics technologies (genomics, transcriptomics, proteomics, and metabolomics). It is possible to produce specialized microbial strains with improved degradation of toxin resistance through genetic engineering. The use of genetically engineered microorganisms (GEMs) in synthetic biology is improving the removal of xenobiotics. With their enhanced capacity to degrade contaminants, GEMs will undoubtedly play a significant role in this field in the future [138]. However, because environmental systems are complicated, it is difficult to comprehend natural microbes, which are necessary for developing desirable synthetic communities. While metagenomics allows for the identification and analysis of complex microbial communities, particularly unculturable organisms, omics methods offer profound insights into microbial metabolism, stress responses, and essential gene expression [139]. When combined, these technologies enable the development of biological treatment methods that are more effective, focused, and robust, significantly expanding their suitability for handling complex industrial wastewater, such as that produced by the explosives manufacturing sector.

More investigation is necessary to achieve remarkable bioremediation improvements by generating new genetically modified strains with strong catabolizing genes. Field studies and a deeper understanding of omics microbial genetics to improve the capacity to degrade explosive contaminants will undoubtedly lead to innovations in this area. This method can offer an integrated process to detoxify the environment when combined with other physical and chemical procedures. As it may be a promising approach for the future, further investigation in this field is warranted. Moreover, the development of bioremediation technologies for full-scale use would be intriguing.

## Figures and Tables

**Table 1:** Common physico-chemical properties of RDX, HMX, and CL-20 [26].

	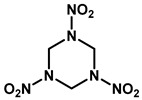	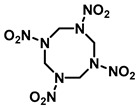	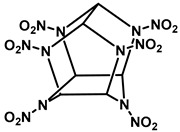
Common Name	RDX	HMX	CL-20
Chemical Formula	C_3_H_6_N_6_O_6_	C_4_H_8_N_8_O_8_	C_6_H_6_N_12_O_12_
Molecular Weight (g/mol)	222.26	296.16	438.19
Melting Point (℃)	205	276–286	260 (with decomposition)
Water Solubility (mg/L) at 25 °C	40.0	6.6	3.6
Octanol/Water Partition Coefficient (log K_w_)	0.90	0.17	1.92
Henry’s Law Constant at 25 °C (atm m^3^/mol)	1.96 × 10^−11^	2.60 × 10^−15^	Not determined
Vapor Pressure at 25 °C (mm Hg)	4.0 × 10^−9^	3.3 × 10^−14^	Not determined
Permissible limit (mg/L) in drinking water	0.002	0.04	Not determined

**Table 2:** Effects of RDX, HMX, and CL-20 on different species.

Compounds	Species	Effects	References
**RDX**	Nematodes (*Caenorhabditis elegans*)	generation of reactive oxygen species, death of germ cells, and a reduction in the number of eggs produced	[32]
	Earthworm (*Eisenia fetida*)	negative impact on reproduction	[33]
	Northern bobwhites (*Colinus virginianus*)	decline in total feed consumption, total egg production, and hen-housed production characteristics	[34]
	Zebrafish	lethally poisoned	[35]
	Fathead minnows	overt toxicity	[36]
	Deer mice (*Peromyscus maniculatus*)	age-dependent toxicity	[37]
**HMX**	Green anoles (*Anolis carolinensis*)	lower hatching success rate	[39]
	Three vertebrate species (rabbit (*Oryctolagus cuniculus*)), amphibians (redbacked salamander (*Plethodon cinereus*)), and reptiles (Western fence lizard (*Sceloporus occidentalis*))	While Salamanders showed no effects at ≤1970 mg/kg HMX, lizards died at high oral dosages, due to gastrointestinal impaction. Rabbits showed neurological symptoms, more sensitive than amphibians and reptiles	[40]
	Northern bobwhites (*Colinus virginianus*)	concentration-dependent reduction in food intake, body mass, and egg production	[41]
	Earthworm (*Eisenia andrei*)	some reproductive consequences, such as the quantity of juveniles and their biomass	[42,43]
**CL-20**	Nematodes and microarthropods	Nematodes remained the same, but microarthropods drastically decreased	[46]
	Earthworm (*Eisenia andrei*)	reproductive toxicity	[47]
	Earthworm (*Eisenia fetida*)	neurotoxicity through dermal contact	[48,49]
	Potworms (*Enchytraeus albidus* and *Enchytraeus crypticus*)	dramatically decreasing adult survival and juvenile production	[50]
	Japanese quail embryos (*Coturnix coturnix japonica*)	produce adverse developmental effects	[51]

**Table 3:** Composition of wastewater contaminated by RDX, HMX, and CL-20.

Wastewater	Parameters	Value	References
**RDX wastewater**	pH	2.0	[18]
	Nitrate (mg/L)	13,869.9	
	COD (mg/L)	6153.6	
	RDX (mg/L)	135.77	
**RDX and HMX wastewater**	pH	1–3	[7,20,58,61,62]
	Acetate (mg/L)	2700–227,000	
	Sulfate (mg/L)	350	
	Ammonium nitrate (mg/L)	200–250	
	Nitrate (mg/L)	400–342,350	
	COD (mg/L)	350–430,000	
	BOD (mg/L)	1238	
	RDX (mg/L)	400–500	
	HMX (mg/L)	200–500	
**CL-20 wastewater**	pH	5.4	[60]
	COD (mg/L)	39,333	
	CL-20 (mg/L)	15.7	
	Ethyl acetate (mg/L)	11,250	
	Chloroform (mg/L)	1120	
	Total nitro compound (mg/L)	65	

**Table 4:** Various remediation methods for RDX, HMX, and CL-20.

Treatment Methods		Compounds	References
**Adsorption**	granular activated carbon	RDX and HMX from polluted groundwater	[65]
	activated carbon fiber (ACF) cloth filled with nanoscale TiO_2_ particles	RDX wastewater	[66]
	mesoporous silicas	HMX	[67]
**Chemical Reduction**	nano-zero-valent iron (nZVIs)	RDX and HMX	[70]
	nZVI@MG (nZVI assembled on magnetic Fe_3_O_4_/reduced graphene oxides composite)	CL-20	[71]
**Advanced Oxidation Processes (AOPs)**	photo-peroxidation and photo-Fenton oxidation	RDX	[74]
	Fenton process	explosive-polluted wastewater	[63]
	UV irradiation used along with hydrogen peroxide and ozone	groundwater from a US Army facility contaminated by explosives	[75]
	direct photolysis, photo-peroxidation, and photo-Fenton oxidation	RDX wastewater	[18]
	photolyzed in a Rayonet photoreactor at 300 nm	CL-20	[24]
	Using nano-TiO_2_ as the photocatalytic catalyst	RDX wastewater	[66]
	UV lamp and TiO_2_	RDX and HMX	[76]
	electro-assisted Fenton method	RDX and HMX from real wastewater	[77]
**Alkaline Hydrolysis**	Alkaline hydrolysis in an aqueous solution (pH 10–12.3)	RDX, HMX, and CL-20	[80]

**Table 5:** Various microbial species for RDX, HMX, and CL-20 degradation.

Compounds	Organism	Pathways and Degradation Products	References
**RDX**	*Gordonia* sp.	NDAB	[88]
	*Janibacter cremeus*	BHNA and MDNA	[89]
	*Shewanella oneidensis* MR-1	nitro reduction	[90]
	*Shewanella putrefaciens* CN32	ammonium, formaldehyde, and nitroso intermediates	[91]
	*Clostridium bifermentans* strain HAW-1	MNX	[92]
	*Clostridium* sp. strain EDB2	Denitration pathway	[14]
	*Gordonia* sp. KTR9	NDAB	[93]
	*Rhodococcus* strains, DN22, 11Y, and A	MEDINA and NDAB	[94]
	*Rhodococcus* strains, YH1, T7, and T9N	NDAB	[86,95,96]
**HMX**	*Clostridium bifermentans* strain HAW-1	MEDINA and the mononitroso derivatives	[99]
	*Planomicrobium flavidum* strain S5-TSA-19	MEDINA and N-methyl-N,N′-dinitromethanediamine	[100]
	*Bacillus* strains (HPB2, HPB3) and *Pseudomonas* (HPB1)	Products not identified	[102]
**CL-20**	*Pseudomonas* sp. ZyL-01	Mechanism not identified	[103]
	*Escherichia coli* nitroreductase	denitration pathway	[104]
	Two enzymes, a diaphorase from *Clostridium kluyveri* and a dehydrogenase from *Clostridium* sp. EDB2	N-denitrohydrogenated products	[105]

## Data Availability

No new data were created or analyzed in this study.

## Abbreviations

The following abbreviations are used in this manuscript:

RDX	Hexahydro-1,3,5-trinitro-1,3,5-triazine
HMX	Octahydro-1,3,5,7-tetranitro-1,3,5,7-tetrazocine
CL-20	2,4,6,8,10,12-Hexanitro-2,4,6,8,10,12-hexaazaisowurtzitane
MNX	Hexahydro-1-nitroso-3,5-dinitro-1,3,5-triazine
DNX	Hexahydro-1,3-dinitroso-nitro-1,3,5-triazine
TNX	Hexahydro-1,3,5-trinitroso-1,3,5-triazine
ROS	Reactive oxygen species
MEDINA	Methylene dinitramine
BHNA	Bis(hydroxymethyl)nitramine
NDAB	4-Nitro-2,4-diazabutanal
TNT	2,4,6-Trinitrotoluene
XO	Xanthine oxidase
MBR	Membrane bioreactor
COD	Chemical oxygen demand
UASB	Up-flow anaerobic sludge bed reactor
AFBR	Anaerobic fluidized bed reactors
FFBR	Fixed film bioreactor
GAC	Granulated activated carbon
FBR	Fluidized bed reactors
ZVI	Zero valent iron
HBIW	Hexabenzylhexaazaisowurtzitane
TADBIW	4,10-Dibenzyl-2,6,8,12-tetraacetyl-2,4,6,8,10,12-hexaazaisowurtzitane
TADAIW	2,6,8,12-Tetraacetyl-2,4,6,8,10,12-hexaazaisowurtzitane
TADFIW	4,10-Diformyl-2,6,8,12-tetraacetyl-2,4,6,8,10,12-hexaazaisowurtzitane
ACF	Activated carbon fiber
ZVINs	Zero-valent iron nanoparticles
PRB	Permeable reactive barrier
AOP	Advanced oxidation processes
TOC	Total organic carbon
TN	Total nitrogen
